# Lipid Droplets in Cancer: New Insights and Therapeutic Potential

**DOI:** 10.3390/ijms27020918

**Published:** 2026-01-16

**Authors:** Shriya Joshi, Chakravarthy Garlapati, Amartya Pradhan, Komal Gandhi, Adepeju Balogun, Ritu Aneja

**Affiliations:** 1 Department of Biology, Georgia State University, Atlanta, GA 30303, USA; shriya.joshi@bms.com (S.J.); amartya.pradhan@emory.edu (A.P.); komaltgandhi@som.umaryland.edu (K.G.); abalogun5@student.gsu.edu (A.B.); 2 Discovery and Development Science, Leads Discovery and Optimization, Bristol Myers Squibb, Cambridge, MA 02141, USA; 3 Molecular & Cell Pharmacology, Alkermes Inc., Waltham, MA 02451, USA; 4 School of Medicine, University of Maryland, Baltimore, MD 21201, USA; 5 School of Health Professions, University of Alabama at Birmingham, Office of the Dean, 1716 9th Avenue South, Birmingham, AL 35294, USA

**Keywords:** lipid droplets (LDs), cancer metabolism, drug resistance, metabolic reprogramming, therapeutic targeting

## Abstract

The progression of neoplastic diseases is driven by a complex interplay of biological processes, including uncontrolled proliferation, enhanced invasion, metastasis, and profound metabolic reprogramming. Among the hallmarks of cancer, as revised by Hanahan and Weinberg, the reprogramming of energy metabolism has emerged as a critical feature that enables cancer cells to meet their heightened bioenergetic and biosynthetic demands. One significant aspect of this metabolic adaptation is the accumulation of lipid droplets (LDs) dynamic, cytoplasmic organelles primarily involved in lipid storage and metabolic regulation. LDs serve as reservoirs of neutral lipids and play a multifaceted role in cancer cell physiology. Their accumulation is increasingly recognized as a marker of tumor aggressiveness and poor prognosis. By storing lipids, LDs provide a readily accessible source of energy and essential building blocks for membrane synthesis, supporting rapid cell division and growth. Moreover, LDs contribute to cellular homeostasis by modulating oxidative stress, maintaining redox balance, and regulating autophagy, particularly under nutrient-deprived or hypoxic conditions commonly found in the tumor microenvironment. Importantly, LDs have been implicated in the development of resistance to cancer therapies. They protect cancer cells from the cytotoxic effects of chemotherapeutic agents by buffering endoplasmic reticulum (ER) stress, inhibiting apoptosis, and facilitating survival pathways. The presence of LDs has been shown to correlate with increased resistance to a variety of chemotherapeutic drugs, although the precise molecular mechanisms underlying this phenomenon remain incompletely understood. Emerging evidence suggests that chemotherapy itself can induce changes in LD accumulation, further complicating treatment outcomes. Given their central role in cancer metabolism and therapy resistance, LDs represent a promising target for therapeutic intervention. Strategies aimed at disrupting lipid metabolism or inhibiting LD biogenesis have shown potential in sensitizing cancer cells to chemotherapy and overcoming drug resistance. In this review, we comprehensively examine the current understanding of LD biology in cancer, highlight studies that elucidate the link between LDs and drug resistance, and discuss emerging approaches to target lipid metabolic pathways to enhance therapeutic efficacy across diverse cancer types.

## 1. Introduction

Metabolic reprogramming is a fundamental way for cancer development and progression [1]. One of the most essential metabolic alterations in cancer is the high metabolic demand and subsequent increase in lipogenesis and lipid metabolism during the tumor growth [1]. One effect of this alteration is an increase in the formation of LDs, which are dynamic organelles that can provide energy via β-oxidation in stressful conditions and provide machinery to produce phospholipid membranes that serve as key signaling molecules [2]. LD biogenesis is an intricate process and various stimuli such as hypoxia, obesity, infection, or extracellular signaling molecules are involved in this process [2,3]. These stimuli alter the expression of various genes that regulate de novo lipid synthesis, induction of extracellular lipid uptake and LD biogenesis. LDs formed by these different stimuli harbor specific lipid content and a set of enzymes that are directly related to lipogenesis, such as Fatty acid synthase and Diacylglycerol acyltransferases, that promote LD accumulation [4,5]. LD homeostasis in cancer cells is regulated by various metabolic enzymes and oncogenes [6]. Lipid metabolic enzymes that are responsible for LD homeostasis include LPCAT2, cytosolic phospholipase 2 (cPLA2), sterol O-acyltransferase 1 (SOAT1), and squalene epoxidase (SQLE) [7]. It has been shown that loss of PTEN and activation of phosphoinositide 3-kinase (PI3K)/AKT/mTOR pathway is associated with increase in LD density. In addition, FOXO3/Sirtuin6 signaling is a negative regulator of lipid biogenesis and thus are involved in depletion of LDs [8]. Cumulatively, these results indicate that tight balance between LD biogenesis and homeostasis is critical for cancer progression.

LDs work in concert with other organelles to carry out important cellular processes including lipid storage, lipid metabolism, energy production, gene regulation, and signal transduction [9]. Due to their dynamic nature, cancer cells harness LDs to sustain their high energy demands. In cancer cells, LD biogenesis is linked to the adaptive response pathways *viz.* oxidative stress resistance, unfolded protein response (UPR) pathway, nutrient stress resistance, evasion of the immune system and the chemoresistance, which are integral to cancer cells [6,10,11]. For instance, LD abundance was shown to be correlated to cancer aggressiveness and cancer cell survival in breast cancer (BC) cell lines [7,12]. Higher LD accumulation was evident in MDA-MB-231, a highly aggressive and malignant TNBC cell as opposed to intermediate and non-malignant, MCF7 and MCF10A BC cell lines, respectively [13,14]. Another data by Mehdizadeh, A. et al. have shown increase in LD accumulation upon treatment of doxorubicin to human colorectal cancer (CRC) cell line, SW480. These data strongly suggest that LD density is positively correlated with tumor aggressiveness, particularly in drug resistance [14]. Thus, there is an unmet need to target LDs in various cancers to improve the therapeutic response of various chemotherapy drugs. In the following sections, we discuss the available methods for LD staining, the role of LDs in cancer growth and drug resistance, and possible therapeutic approach to target them particularly in BC, CRC, Prostate cancer (PCa) and pancreatic ductal adenocarcinoma (PDAC).

## 2. Molecular Composition of LDs in Cancer Cells

LDs are highly with a distinct architecture and specialized composition. Structurally, LDs consist of a hydrophobic core of neutral lipids—primarily triacylglycerols (TAGs) and cholesteryl esters—surrounded by a phospholipid monolayer that maintains organelle integrity and mediates interactions with other cellular compartments. The surface monolayer is enriched in phosphatidylcholine and phosphatidylethanolamine, which stabilize the droplet and regulate lipid exchange [15]. Beyond lipids, LDs harbor a diverse set of proteins that govern their biogenesis, turnover, and signaling functions. These include structural proteins such as perilipins (PLIN1–PLIN5), adipophilin (PLIN2), and enzymes involved in lipid metabolism like diacylglycerol acyltransferases (DGAT1/2), adipose triglyceride lipase (ATGL), fatty acid synthase (FASN), and stearoyl-CoA desaturase 1 (SCD1). Proteomic studies have revealed that LDs also recruit stress-response proteins, chaperones, and even signaling molecules, suggesting roles beyond simple lipid storage [16].

In cancer cells, LD composition is often reprogrammed to meet the demands of rapid proliferation and therapy resistance. Lipidomic analyses indicate an enrichment of unsaturated fatty acids and cholesteryl esters, which enhance membrane fluidity and support oncogenic signaling. Elevated levels of LD-associated proteins such as PLIN2, PLIN3, FASN, and SCD1 correlate with aggressive phenotypes, metastatic potential, and poor patient survival, underscoring their utility as biomarkers and therapeutic targets. Furthermore, LDs may serve as reservoirs for bioactive lipids that modulate ER stress, oxidative stress, and ferroptosis sensitivity, linking their composition directly to cancer cell adaptability under chemotherapeutic pressure [17].

## 3. LDs as Drivers of Cancer Cell Invasion and Aggressiveness

The accumulation of LDs within cancer cells is increasingly recognized as a critical factor in promoting tumor aggressiveness, including enhanced invasion, migration, and proliferation. LDs serve not only as lipid storage organelles but also as metabolic hubs that support the energetic and biosynthetic demands of rapidly dividing and migrating cancer cells. Their role in cancer progression has been particularly well-documented in PDAC, BC, and PCa.

In PDAC, Rozeveld et al. (2021) [18,19] demonstrated that inhibition of DGAT1/2—enzymes essential for the final step of triglyceride synthesis and LD formation—significantly reduced lipid-induced invasion in mKPC cells. This finding underscores the functional importance of LD biogenesis in facilitating the invasive behavior of PDAC cells. By limiting the packaging of lipids into LDs, the cells were deprived of a key resource for membrane synthesis and energy production, thereby impairing their invasive potential.

Breast cancer cells exhibit a similar dependency on LDs for invasion. Nisticò et al. (2021) [19] found that pharmacological inhibition of DGAT2 in MCF7 cells led to a marked reduction in cancer cell invasion, although cell migration remained largely unaffected. This suggests that LDs may specifically support the invasive machinery—such as matrix metalloproteinase activity or cytoskeletal remodeling—without necessarily influencing motility pathways. The selective impact on invasion highlights the nuanced role of LDs in modulating distinct aspects of cancer cell behavior.

In PCa, Nardi et al. (2019) [20] reported that DGAT1 is significantly overexpressed in malignant cells compared to normal prostate tissue. Inhibition of DGAT1 not only reduced LD formation but also suppressed cell proliferation, migration, and invasion. These findings suggest that LDs contribute to multiple facets of tumor progression, likely by supplying lipids for membrane biosynthesis, generating ATP through β-oxidation, and buffering oxidative stress.

Mechanistically, LDs may facilitate cancer aggressiveness by serving as lipid reservoirs that support membrane expansion during cell division and invasion, by modulating signaling pathways involved in epithelial-to-mesenchymal transition (EMT), and by maintaining redox homeostasis under metabolic stress. Their dynamic nature allows cancer cells to rapidly adapt to fluctuating environmental conditions, such as hypoxia or nutrient deprivation, which are common in the microenvironment of the tumor.

In addition to promoting tumor progression, LDs also play a protective role against therapeutic stress. They have been shown to mitigate endoplasmic reticulum (ER) stress, inhibit apoptosis, and contribute to chemoresistance—topics that will be explored in the following section. Together, these findings position LDs as central players in both the aggressiveness and treatment resistance of cancer, making them attractive targets for therapeutic intervention.

## 4. Cancer Cells Safeguard Themselves from Chemotherapeutic Agents by Accumulating LDs

Cancer cells often combat metabolic and cellular stress by increasing their LD content and thereby maintain the cell homeostasis [21]. LDs have emerged as critical players not only in cancer metabolism and progression but also in mediating resistance to chemotherapy. One of the mechanisms by which LDs contribute to drug resistance is through the sequestration of hydrophobic chemotherapeutic agents, thereby impairing their cytotoxic efficacy. This phenomenon has been particularly well-documented in breast cancer cells. In a study by Schlaepfer et al. (2012) [21], it was demonstrated that the chemotherapeutic drug docetaxel, which is highly hydrophobic, can be sequestered within LDs. This intracellular compartmentalization of the drug led to a marked reduction in its ability to induce apoptosis, ultimately resulting in decreased sensitivity of the cancer cells to docetaxel treatment. The lipophilic nature of docetaxel facilitates its diffusion across the plasma membrane but also predisposes it to accumulate in lipid-rich environments such as LDs, effectively isolating it from its intracellular targets.

This concept is further supported by the work of Dubey et al. (2020) [22], who investigated the intracellular distribution of Lasonolide-A (LasA), a hydrophobic prodrug, and its active, charged metabolite Lasonolide-F (LasF). Their study in Hap1 cells revealed that LasA preferentially localized to LDs, whereas LasF, due to its charged nature, remained predominantly in the cytoplasm. This differential localization underscores the role of LDs as selective reservoirs for hydrophobic compounds, which may shield cancer cells from the full impact of chemotherapeutic agents. The sequestration of drugs in LDs not only reduces their effective concentration in the cytosol but also delays or prevents the activation of apoptotic pathways, thereby enhancing cancer cell survival under therapeutic stress [22].

Persistent ER stress activates the unfolded protein response (UPR) through three sensors—pERK, IRE1α, and ATF6—to restore proteostasis. Initially, pERK phosphorylates eIF2α to reduce global translation while inducing ATF4, IRE1α splices XBP1 mRNA to enhance chaperone expression, and ATF6 translocates to the Golgi for activation. Under conditions of prolonged, unmitigated ER stress, adaptive signaling shifts toward apoptosis via CHOP induction and ER-resident caspases. Caspase-12, anchored on the ER membrane, is cleaved by calcium-dependent calpains following ER calcium efflux, linking ER dysfunction to cell death. This pathway is distinct from mitochondrial apoptosis and represents a hallmark of ER stress-mediated cell death. Cancer cells with elevated LDs exhibit enhanced resistance to reactive oxygen species (ROS) and lipotoxicity, two major contributors to ER stress. LDs act as metabolic buffers by sequestering excess fatty acids and toxic lipids, stabilizing ER membrane integrity, and reducing calcium dysregulation, thereby limiting caspase-12 activation. This buffering delays the transition from adaptive UPR to apoptotic signaling, enabling survival under chemotherapeutic stress such as gemcitabine. Mechanistically, LD-mediated protection intersects with KRAS-driven lipid metabolic reprogramming, which promotes LD biogenesis and sustains ER homeostasis. Therapeutically, targeting LD formation (e.g., DGAT1/2 inhibition) or enhancing ER stress execution pathways (e.g., CHOP or caspase activation) could restore ER stress-induced apoptosis and overcome drug resistance [23]. Myriads of available literatures suggests that there is an association between increased number of LDs and acquired drug resistance in cancer. These findings suggest that LDs serve as a protective niche within cancer cells, capable of modulating drug distribution and efficacy. As such, targeting LD formation or disrupting their capacity to sequester chemotherapeutic agents represents a promising strategy to overcome drug resistance and improve treatment outcomes in cancer therapy.

In the following section, we discuss this association particularly in BC, PDAC, PCa and CRC.

### 4.1. Role of LDs in Drug Resistance of BC

Several studies have identified that aggressive BC cells have higher numbers of intracellular LDs. A study by Abramczyk, H., et al. (2015) [13], suggest that LD abundance is correlated with degree of aggressiveness in BC cell lines. They found a higher LD accumulation in highly aggressive.

MDA-MB-231 cells compared to moderately aggressive MCF-7 cells and least LD accumulation was evident in nonmalignant MCF10A BC cells [13]. The higher lipid content in malignant breast cancer cells may be due to increased fatty acid and phospholipid synthesis, which enhances cell proliferation and prevents cells from undergoing apoptosis. Upon chemotherapy, BC cells often increase import of fatty acids to either generate energy through β-oxidation or store them into LDs [24,25]. Our in-vitro data strongly suggests a significantly higher accumulation of LDs upon treatment with Doxorubicin in triple negative (TN) BC cells. Tian et al. (2019) [26] conducted a retrospective study assessing the serum lipid levels in patients with BC at specific intervals upon chemotherapy and compared the effects of chemotherapy agents (anthracycline, taxane, and anthracycline and taxane combination regimen) on serum lipid profiles. Their results revealed an increase in triglycerides (TG), total cholesterol (TC), and low-density lipoprotein cholesterol (LDL-C) serum lipids levels upon chemotherapy. In contrast, homocysteine (HCY) and high-density lipoprotein cholesterol (HDL-C) decreased markedly after chemotherapy. These levels were significantly altered by six months post chemotherapy. Based on the results of this and other studies, it is evident that there is an association between the chemotherapy agents and lipid profile change among patients with BC. Specific metabolic pathways for these alterations in lipid profiles upon chemotherapy in BC are not fully understood, and thus, further investigation into the mechanisms is highly warranted.

### 4.2. Role of LDs in Drug Resistance of PDAC, PCa, and CRC

It is increasingly acknowledged that atypical de novo lipid synthesis and reprogrammed lipid metabolism are both associated with the development and progression of PDAC. Oncogenic KRAS not only drives proliferative signaling but also orchestrates metabolic reprogramming that favors lipid storage. Mechanistically, KRAS modulates the activity of hormone-sensitive lipase (HSL), reducing lipolysis and promoting LD accumulation. These LDs act as reservoirs for fatty acids and cholesterol, which buffer cells against lipotoxicity and endoplasmic reticulum (ER) stress. Under chemotherapeutic pressure—such as gemcitabine treatment—this buffering capacity prevents ER stress–induced apoptosis, thereby enabling survival. In essence, KRAS-driven LD formation creates a metabolic shield that sustains cellular homeostasis and contributes to chemoresistance in PDAC [27]. Targeting this axis—either by disrupting KRAS signaling or interfering with LD formation and lipid storage—could offer a promising strategy to enhance the efficacy of gemcitabine and other chemotherapeutic agents in KRAS-mutant pancreatic cancers [18,28].

A study by Saber B.T. et al. (2017) [29], identified lipid metabolism is most significantly correlated with poor gemcitabine response in patients with PDAC. They observed a significant correlation of increase in fatty acid synthase (FASN) expression and poor response to gemcitabine treatment in PDAC. Emerging evidence indicates that cancer cells frequently overexpress low-density lipoprotein receptors (LDLR), which enhances the uptake of circulating low-density lipoproteins (LDL) a major source of cholesterol. This increased LDL uptake contributes to elevated intracellular cholesterol levels and promotes the formation ofLDs, as imported LDL particles can fuse and be stored within these organelles. The accumulation of cholesterol-rich LDs not only supports membrane biosynthesis and energy storage but also plays a role in protecting cancer cells from therapeutic stress. In PDAC, Guillaumond et al. (2015) [30] demonstrated that blocking cholesterol uptake sensitizes cancer cells to chemotherapeutic agents. Their findings suggest that cholesterol metabolism, particularly the uptake and storage of LDL-derived cholesterol in LDs, contributes to the chemo resistant phenotype of PDAC. By buffering endoplasmic reticulum stress and maintaining lipid homeostasis, LDs may enable cancer cells to survive under the cytotoxic pressure of chemotherapy. These insights underscore the potential of targeting cholesterol uptake and LD formation as a therapeutic strategy to overcome drug resistance in PDAC and possibly other malignancies characterized by dysregulated lipid metabolism [30,31]. On similar lines, we observed a significant increase in LD density upon gemcitabine treatment to various PDAC cell lines. Current evidence indicates a strong correlation between LD accumulation and chemoresistance in PDAC; however, causality remains to be fully established. While KRAS-driven metabolic reprogramming and LD dynamics appear to support survival under therapeutic stress, these observations are primarily associated. Functional studies are required to confirm whether LD modulation directly mediates resistance or represents an adaptive response.

Altered lipid metabolism is a hallmark of PCa. In PCa, FASN is upregulated leading to LD accumulation. Androgen receptor (AR), an important gene regulating PCa progression is involved in regulating the cellular uptake of exogenous lipids and enzymes involved in de novo lipogenesis [32,33]. The fatty acids taken up or synthesized by PCa cells can then be stored in LDs [33]. Studies show that lipid accumulation in PCa is closely linked to development of castration resistant prostate cancer (CRPC) and Enzalutamide or Abiraterone resistance. Kaini R. et al. (2012) [34] found PCa cells metabolize the lipids stored in LDs, helping the cells survive androgen deprivation therapy. Our in vitro data in PC3 cells treated with docetaxel showed higher accumulation of LDs than the untreated PCa cells. A very recent study by Zhou L. et al. (2021) [35] indicated that acyl-CoA synthetase short chain family member 3 (ACSS3) is involved in degradation of LDs, and thereby improves therapy response in PCa. Mechanistically, ACSS3 represses LD deposits by regulating the stability LD coat protein perilipin 3 (PLIN3).

LD accumulation is disadvantageous to the treatment of CRC. Data by Cotte et al. (2018) [36], showed that LD accumulation is tightly linked with 5-flurouracil (5-FU) drug resistance in CRC. They have shown that the treatment of 5-FU and oxaliplatin promoted LD accumulation in CRC. Lysophosphatidylcholine acyltransferase 2 (LPCAT2) induces the accumulation of LDs, leading to chemoresistance in CRC. In another study, it was reported that the chemotherapy agents altered the lipid metabolism and fatty acid distribution in CRC by releasing LDs [14]. In similar line, our data also revealed accumulation of LDs upon treatment with 5-FU in CRC cells. LDs may also contribute to chemoresistance by modulating sphingolipid metabolism, particularly through the sequestration of acylceramides. In CRC cells, ceramides—bioactive sphingolipids known to promote apoptosis—can be enzymatically converted into acylceramides, which are then stored in LDs. This metabolic shift reduces the intracellular levels of pro-apoptotic ceramides, thereby enhancing cancer cell survival under chemotherapeutic stress.

Senkal et al. (2017) [37] demonstrated that the conversion of ceramides to acylceramides, followed by their storage in LDs, was associated with resistance to 5-fluorouracil (5-FU) in CRC cells. Importantly, when the synthesis of acylceramides was inhibited, ceramide levels increased, leading to the induction of apoptosis. These findings suggest that LDs serve as protective reservoirs, sequestering acylceramides and preventing the accumulation of cytotoxic ceramides. By buffering ceramide-induced stress, LDs may thus play a critical role in promoting chemoresistance. This mechanism highlights the broader role of LDs in regulating lipid signaling pathways that influence cell fate decisions. Targeting enzymes involved in ceramide metabolism or disrupting LD formation could represent a novel therapeutic strategy to sensitize CRC cells to 5-FU and potentially other chemotherapeutic agents [37].

In summary, all these results strongly indicate that there is an association of LDs with drug resistance in various cancers. However, the precise mechanism by which LDs cause drug resistance in these cancers needs further thorough investigation.

## 5. LD Dynamics in Cancer: Mechanistic Insights and Therapeutic Strategies

LDs are not merely passive lipid storage sites, but dynamic organelles involved in energy homeostasis, membrane biosynthesis, and cellular stress responses. Their formation, growth, and degradation are tightly regulated by a network of signaling pathways, transcriptional regulators, and post-transcriptional mechanisms. The PI3K/AKT/mTOR pathway is a master regulator of cell growth and metabolism. Activation of PI3K leads to AKT phosphorylation, which subsequently activates mTORC1. mTORC1 enhances lipid synthesis by upregulating SREBP1, a transcription factor that promotes the expression of fatty acid synthase (FASN) and acetyl-CoA carboxylase (ACC), both essential for LD formation [38]. In contrast, AMPK acts as a cellular energy sensor. Under low-energy conditions, AMPK inhibits ACC and SREBP1, thereby reducing fatty acid synthesis and LD accumulation. It also promotes fatty acid oxidation, counterbalancing lipid storage [38]. SREBP1 and SREBP2 are key transcription factors that regulate genes involved in fatty acid and cholesterol biosynthesis, respectively. Their activation is crucial for LD biogenesis, especially under oncogenic or metabolic stress [38]. PPARγ and C/EBPα/β are central transcription factors that regulate adipogenesis and lipid metabolism, ensuring lipid storage and metabolic flexibility. In cancer, these pathways may be repurposed to sustain rapid proliferation and stress tolerance. Beyond its metabolic role, C/EBPβ may also influence tumor aggressiveness by activating genes involved in mitotic integrity, such as KIFC1, in AR-negative triple-negative breast cancer. Interestingly, C/EBPβ has also been implicated in prostate cancer progression, where it may interact with androgen receptor (AR) signaling to modulate tumor growth and therapy response. Similarly, PPARα, which controls fatty acid oxidation, may provide energy and redox balance to support survival under therapeutic stress. Together, these regulators may form an interconnected network: PPARγ–C/EBPα/β programs lipid supply, PPARα fuels oxidative metabolism, and C/EBPβ potentially links these metabolic adaptations to proliferative fitness and AR-related pathways. This possible convergence suggests that metabolic rewiring and mitotic stability are not isolated processes but may cooperate to drive aggressive phenotypes, highlighting a rationale for strategies that co-target lipid metabolism and cell division pathways in AR-negative TNBC and AR-driven prostate cancer [39,40,41]. Non-coding RNAs also play a significant role in LD regulation. For instance, miR-33a/b suppresses genes involved in lipid efflux and fatty acid oxidation, favoring lipid accumulation in LDs. Long non-coding RNAs such as H19 and circular RNAs like CDR1as modulate lipid metabolism by interacting with miRNAs or transcription factors, influencing LD dynamics and contributing to chemoresistance. Post-translational modifications (PTMs) of LD-associated proteins are critical for regulating LD function. Proteins such as perilipins are regulated by phosphorylation, ubiquitination, and acetylation. For example, PKA-mediated phosphorylation of perilipin-1 facilitates lipolysis, while ubiquitination of ATGL (adipose triglyceride lipase) targets it for degradation, reducing lipid breakdown. LDs also form contact sites with the ER, mitochondria, and lysosomes. These interactions facilitate lipid exchange, energy production, and autophagic degradation of LDs (lipophagy), which are essential for maintaining lipid homeostasis and adapting to stress. Lipid droplet-associated proteins such as perilipins, ATGL, and hormone-sensitive lipase (HSL) are central to the structural integrity and metabolic function of LDs [38]. Perilipins coat the LD surface and regulate access to lipases, while ATGL and HSL mediate the hydrolysis of stored triglycerides. Lipid signaling molecules including diacylglycerol (DAG), ceramides, and sphingolipids influence LD dynamics by modulating lipid synthesis and degradation pathways. These bioactive lipids are involved in apoptosis, proliferation, and inflammation, linking LD biology to broader cellular signaling networks. Metabolic flux regulators such as ACC and FASN control the flow of metabolites through lipid biosynthesis and oxidation pathways. Their activity is modulated by upstream signaling cascades like PI3K/AKT/mTOR and AMPK, which directly impact LD formation and utilization [38]. The PI3K/AKT/mTOR pathway not only drives anabolic growth and lipid synthesis but also actively suppresses autophagy, including lipophagy, the selective degradation of LDs. Mechanistically, PI3K activation generates PIP3, recruiting AKT to phosphorylate downstream targets that activate mTORC1. Activated mTORC1 phosphorylates ULK1 at Ser757, preventing its interaction with AMPK and thereby blocking autophagy initiation. In addition, mTORC1 inhibits TFEB nuclear translocation, reducing lysosomal biogenesis and autophagic flux. This dual blockade stabilizes LD pools by preventing their clearance, reinforcing lipid storage and buffering ER stress. In PDAC, KRAS-driven lipogenesis combined with mTOR-mediated lipophagy suppression creates a lipid-rich environment that supports chemoresistance by providing membrane precursors, maintaining redox balance, and limiting ER stress-induced apoptosis. Therapeutically, restoring lipophagy through mTOR inhibition or TFEB activation could dismantle LD reserves, increase ER stress, and sensitize tumors to chemotherapy. Combining mTOR inhibitors with lipogenesis blockers (e.g., DGAT1/2 inhibitors) may synergistically reduce LD burden and overcome drug resistance [42,43]. Autophagy-related proteins such as LC3 and p62 are involved in the selective degradation of LDs through lipophagy. This process is crucial for maintaining cellular lipid balance and is influenced by nutrient availability and metabolic stress [38]. Mitochondrial dynamics, including fission and fusion processes, are essential for maintaining mitochondrial function and energy production. Mitochondria interact with LDs to facilitate lipid exchange and oxidation, which is important for cellular energy homeostasis and adaptation to metabolic stress.

Targeting LD biogenesis and lipid metabolic pathways has emerged as a promising strategy to overcome chemoresistance in PDAC and other cancers. Approaches such as DGAT1/2 inhibitors to block triglyceride synthesis, FASN inhibitors like TVB-2640 to reduce de novo lipogenesis, and mTOR inhibitors to restore lipophagy have shown encouraging preclinical results. These interventions aim to dismantle LD reserves, amplify ER stress, and sensitize tumors to chemotherapy. However, translating these findings into clinical success remains challenging. TVB-2640, the first-in-class FASN inhibitor, demonstrated manageable safety and modest efficacy in early-phase trials, but adverse effects including mucositis, dry eye, and skin toxicity were common, and long-term tolerability is uncertain. Off-target effects are a major concern because enzymes such as FASN and DGAT are expressed in normal tissues, raising the risk of systemic metabolic disruption. Tumor heterogeneity further complicates patient selection, as not all cancers exhibit equal dependency on lipid metabolism, and reliable biomarkers of response are still lacking. Moreover, adaptive resistance mechanisms, such as increased lipid scavenging or activation of alternative metabolic pathways, may limit the durability of LD-targeted therapies. To enhance therapeutic impact, future strategies should integrate LD-targeted agents with complementary approaches such as autophagy modulators or ferroptosis inducers and incorporate biomarker-driven patient stratification. Rational combinations—for example, mTOR inhibitors paired with DGAT blockers—may synergistically reduce LD burden and restore chemosensitivity, but these concepts require rigorous clinical validation before they can be translated into practice [44,45,46]. Lipid-degrading small molecules that induce ferroptosis—a form of iron-dependent cell death—are also being developed. These agents exploit the vulnerability of drug-resistant cancer cells that accumulate iron and polyunsaturated lipids in lysosomes and LDs [47]. These trials reflect a growing interest in exploiting the metabolic dependencies of cancer cells, particularly those involving lipid storage and utilization.

Lipid metabolism not only supports tumor growth but also modulates the tumor immune microenvironment. This has led to the development of combination therapies that integrate LD-targeting agents with other treatment modalities. Tumor-associated macrophages (TAMs), regulatory T cells (Tregs), and myeloid-derived suppressor cells (MDSCs) thrive in lipid-rich environments. Inhibiting lipid uptake or synthesis in these cells can reduce their immunosuppressive activity. For example, combining FASN inhibitors with anti-PD-1/PD-L1 checkpoint inhibitors has shown synergistic effects in preclinical models by enhancing T cell infiltration and activity [47]. LDs protect cancer cells from radiation-induced oxidative stress. Agents that deplete LDs or inhibit their formation can sensitize tumors to radiation. Preclinical studies in breast and prostate cancer models have demonstrated that LD-targeting drugs enhance the efficacy of radiotherapy by increasing lipid peroxidation and impairing DNA repair. The PI3K/AKT/mTOR pathway, a key regulator of lipid metabolism, is frequently activated in cancer. Combining mTOR inhibitors with FASN inhibitors has shown promise in overcoming resistance in triple-negative breast cancer and prostate cancer. These combinations disrupt both signaling and metabolic support systems of tumor cells [47]. Emerging strategies also include the use of mRNA–lipid nanoparticles (LNPs) to deliver therapeutic payloads that modulate lipid metabolism or immune responses. Clinical trials are evaluating mRNA–LNPs in combination with immune checkpoint inhibitors like durvalumab, aiming to enhance antigen presentation and immune activation [48,49]. Together, these interconnected pathways and molecular players underscore the central role of LDs in cancer metabolism, survival, and therapy resistance. Understanding these mechanisms opens new avenues for targeting LDs in cancer treatment, offering promising strategies to overcome drug resistance and improve therapeutic outcomes.

Future research should focus on elucidating the precise molecular mechanisms governing LD dynamics in various cancer types. This includes identifying novel regulators and understanding their interactions with existing pathways. Additionally, the development of advanced imaging techniques and biomarkers for LDs will enhance our ability to monitor and target these organelles in clinical settings. Exploring combination therapies that target LDs alongside conventional treatments may provide synergistic effects, improving patient outcomes. Ultimately, a deeper understanding of LD biology will pave the way for innovative therapeutic strategies in cancer treatment.

## 6. Diagnostic and Prognostic Potential of LDs in Cancer

LDs are dynamic lipid-rich structures that contribute to cellular homeostasis and hold significant promises for cancer-related diagnostics and prognostics. Their accumulation in various tumor types reflects underlying metabolic reprogramming, often driven by oncogenic signaling pathways such as PI3K/AKT/mTOR. Elevated LD content has been observed in aggressive cancers including glioblastoma, breast, liver, and prostate malignancies, where it correlates with enhanced lipid biosynthesis and storage. Importantly, elevated expression of LD associated proteins—such as perilipins (PLIN2/PLIN3), adipophilin, FASN, and stearoyl-CoA desaturase 1 (SCD1)—has been correlated with higher tumor grade, increased metastatic potential, and poorer patient survival, supporting their utility as biomarkers for disease progression and therapeutic response [50,51]. (Beyond their metabolic roles, LDs also reflect the tumor microenvironment, particularly under hypoxic or nutrient-deprived conditions, which are known to promote therapy resistance. In some cancers, LD accumulation is associated with cancer stem cell phenotypes, further underscoring their relevance in tumor initiation and recurrence (lipid degrading small molecules kills cancer) [1]. These findings position LDs not only as passive indicators of metabolic stress but as active participants in tumor biology, with potential for clinical application in stratifying patients and guiding treatment decisions. Recent advances in imaging technologies have significantly enhanced our ability to monitor LDs in situ. Label-free techniques such as coherent anti-Stokes Raman scattering (CARS) and stimulated Raman scattering (SRS) microscopy enable real-time visualization of LDs based on their unique vibrational signatures, allowing for the differentiation of lipid species within tumors (2) (LDs and flux). Super-resolution microscopy and fluorescence lifetime imaging microscopy (FLIM) provide nanoscale insights into LD dynamics and their interactions with organelles such as mitochondria and the endoplasmic reticulum. Furthermore, the development of LD-targeted contrast agents for MRI and PET imaging is paving the way for non-invasive tracking of lipid metabolism in vivo. Artificial LDs are also being engineered as multifunctional platforms for drug delivery and imaging enhancement, offering targeted approaches for tumors with high LD content [52]. In conclusion, LDs represent a promising frontier in cancer diagnostics and prognostics. Their abundance, composition, and associated proteins offer valuable insights into tumor metabolism, aggressiveness, and treatment response. As imaging technologies continue to evolve, the clinical utility of LDs as biomarkers is likely to expand, enabling more precise and personalized cancer care.

Looking ahead, future research should focus on standardizing LD quantification methods and validating LD-associated biomarkers across diverse cancer types and patient cohorts. Integrating LD imaging with multi-omics approaches could uncover novel regulatory networks and therapeutic vulnerabilities. Additionally, the development of LD-targeted probes and theranostic agents may open new avenues for real-time monitoring and intervention, ultimately enhancing the precision and efficacy of cancer treatment.

## 7. Approaches to Target LDs to Improve Chemotherapy Response

It is now well-established that altered lipid metabolism is associated with resistance to conventional chemotherapies and targeted therapies in several cancers. Lipid metabolic reprogramming includes both changes in de novo lipogenic synthesis and/or lipolytic pathway. Cancer cell resistance is related to the upregulation of lipogenic or lipolytic enzyme expression. Thus, targeting the metabolic pathways specifically the lipid metabolism is an attractive alternative to improve chemotherapy response in various cancers. Recently we have investigated the role of monoethanolamine (Etn), a lipid-based formulation in modulating the metabolism in PCa [53,54]. Interestingly our data have revealed that Etn effectively reduces the LD accumulation in PCa and thereby leading to apoptosis in PCa. We believe that Etn, in combination with other conventional chemotherapy drugs used for PCA, could benefit by reducing the LD accumulation and improve the therapy response in PCa. Various studies have shown increased expression of FASN is responsible for accumulation of LDs. Thus, targeting FASN, a crucial enzyme catalyzing the synthesis of endogenous long chain fatty acid, with its inhibitors could be a viable alternative in lowering the LDs and thereby improving the therapy response in various cancers. Various genes are associated with LD genesis, as well as degradation. Through genomic, proteomic, and metabolomic profiling of LDs could essentially give more clues about their cargo that is responsible for drug resistance in cancer and could open new avenues to target LDs.

Emerging studies have highlighted the critical role of cancer-associated adipocytes (CAAs) within the tumor microenvironment in modulating cancer metabolism and therapy resistance. CAAs contribute to tumor progression by supplying exogenous lipids and reprogramming lipid metabolic pathways in cancer cells. For instance, local adipocytes have been shown to transfer fatty acids to ovarian cancer cells, leading to increased lipid droplet (LD) accumulation and enhanced tumor cell survival. Obesity, which is associated with elevated adipose tissue mass and altered adipocyte function, has been linked to a higher incidence and poorer prognosis in several cancers, including breast cancer (BC), pancreatic ductal adenocarcinoma (PDAC), prostate cancer (PCa), and colorectal cancer (CRC). In a comparative study, Balaban et al. found that adipocytes derived from obese individuals released significantly more fatty acids than those from non-obese individuals, thereby providing a greater lipid supply to adjacent cancer cells. This enhanced lipid transfer may contribute to increased LD formation and metabolic flexibility in tumor cells. In addition to supplying lipids, adipocyte-secreted factors have been shown to activate oncogenic signaling pathways. For example, in melanoma, adipocyte-derived signals were found to activate the AKT pathway, which in turn influenced lipid metabolism and conferred resistance to radiotherapy. Notably, overactivation of the AKT pathway has also been observed in other malignancies, including PDAC, suggesting a broader relevance of this mechanism. Furthermore, CAAs have been implicated in protecting cancer cells from the cytotoxic effects of chemotherapy. Studies have demonstrated that CAAs can shield BC, PDAC, and CRC cells from anti-cancer drugs, potentially by enhancing lipid storage and buffering metabolic stress. Although the precise mechanisms by which CAAs modulate chemoresistance remain to be fully elucidated, these findings underscore the importance of the tumor microenvironment in shaping therapeutic responses. Targeting CAAs or their metabolic interactions with cancer cells—particularly in combination with conventional therapies—may offer a novel strategy to reduce LD accumulation, disrupt lipid metabolism, and overcome drug resistance in solid tumors [22,55,56,57,58,59]. Several studies have showed effectiveness of active food components in reducing the LDs and thereby improving drug response in cancers [60,61]. Particularly, a study by Liang Y.S et al. (2018) [61], has revealed that Genistein and daidzein, isoflavonoids abundant in soya beans, effectively inhibit the LD accumulation and thereby induces apoptosis in CRC. Natural compounds such as epigallocatechin-3-gallate (EGCG), curcumin, and resveratrol have been widely studied for their ability to modulate lipid metabolism and reduce LD accumulation in cancer cells. EGCG, the major catechin in green tea, has shown preclinical efficacy by inhibiting fatty acid synthase, reducing LD formation, and inducing apoptosis through oxidative stress and ER stress pathways. However, most evidence originates from in vitro or animal models, often using concentrations that are not clinically achievable. Clinical trials investigating EGCG have reported poor bioavailability, rapid metabolism, and inconsistent pharmacokinetics, limiting its therapeutic potential. Furthermore, mechanistic studies in human cancers remain incomplete, with gaps in understanding how EGCG interacts with lipid metabolic regulators such as PPARs, SREBF1, and autophagy-related pathways. These limitations underscore the need for rigorous translational research, including dose optimization, formulation strategies to improve bioavailability, and mechanistic validation in patient-derived models. While natural products offer an attractive, low-toxicity approach, their integration into standard cancer therapy requires overcoming these pharmacological and mechanistic barriers [60,62,63]. Thus, supplement of such natural food products along with chemotherapy might prove beneficial in reducing the LD content and thereby improving the drug response in cancers. In summary, in depth knowledge of LD content, molecular mechanisms associated with its generation and degradation, is vital to design therapeutic alternatives that can effectively target LDs and improve the course of disease patients with cancer.

## 8. Available Methods for LD Staining

LD dynamics play a crucial role in cancer progression. Thus, to understand their morphology, mechanism of formation, and degradation, reliable imaging tools and staining protocols for visualization of LDs are emerging. Label free approaches for the visualization of cellular LDs include conventional transmission electron microscopy [64], direct organelle mass spectrometry [65], Raman microscopy [13], and coherent anti-Stokes Raman scattering microscopy [66]. Even though these advanced techniques allow the study of biophysics of LDs, their major demerits are use of fixatives, complex sample preparation and data analysis. Thus, these methods do not present a possibility of studying real time dynamics of LDs. On the contrary, fluorescence-based imaging techniques are more powerful and highly sensitive to study complex biological processes of LDs in a real time settings [38]. Lipid droplet visualization commonly uses lipophilic dyes such as Sudan III and Oil Red O (non-fluorescent diazo dyes) for colorimetric staining, and fluorescent probes like Nile Red and BODIPY 493/503 for imaging. Although fluorescent dyes are widely used, they present challenges including spectral crosstalk, limited photostability, small Stokes shifts, and high background noise. To overcome these limitations, use of fluorescent probes is an emerging alternative to visualize LDs. Fluorescent probes include blue emitting LD probes, green emitting LD probes, orange emitting LD probes, and red to near infra-red emitting LD probes (Table 1) [38,67]. In summary, various approaches to visualize LDs are developing and these will help to better understand the dynamics of LDs in cancer.

## 9. Concluding Remarks and Prospects

Lipids are involved in cancer cell proliferation, migration, and survival of cancer cells upon genotoxic stress. Increased LD accumulation leads to the inhibition of caspase related activation, therapy leading to lower apoptosis and higher survival of cancer cells. LDs contribute to overcoming cellular stress in cancer and thereby maintain their homeostasis. Chemotherapy induces accumulation of LDs. Chemotherapy regimen in combination with inhibitors that reduce LD accumulation, may provide a more efficient solution to drug resistance in various cancers. Specific inhibitors against LD biogenesis or the utility of LD as biomarkers in certain cancers could open a new class of therapeutics in cancers.

Despite the growing promise of lipid droplet (LD)-targeted therapies in oncology, several biological and clinical challenges must be addressed to ensure safe and effective application. A major concern is systemic toxicity and off-target effects. LDs are not exclusive to cancer cells—they are essential for lipid storage, energy homeostasis, and protection against lipotoxicity in nearly all eukaryotic cells. Global inhibition of LD formation or lipid metabolism could disrupt normal physiology, particularly in metabolically active tissues such as the liver and adipose tissue, potentially leading to hepatic steatosis, immune dysregulation, or metabolic syndrome.

Another challenge is the lack of specificity in current LD-targeting agents. Many inhibitors, such as those targeting fatty acid synthase (FASN) or carnitine palmitoyltransferase 1 (CPT1), act on core metabolic pathways shared by malignant and non-malignant cells, narrowing the therapeutic window and increasing adverse effects. Compounding this issue is the metabolic plasticity of cancer cells, which enables adaptation through alternative pathways such as lipid uptake, autophagy, or glycolysis—contributing to therapeutic resistance and relapse.

**Figure 1:**
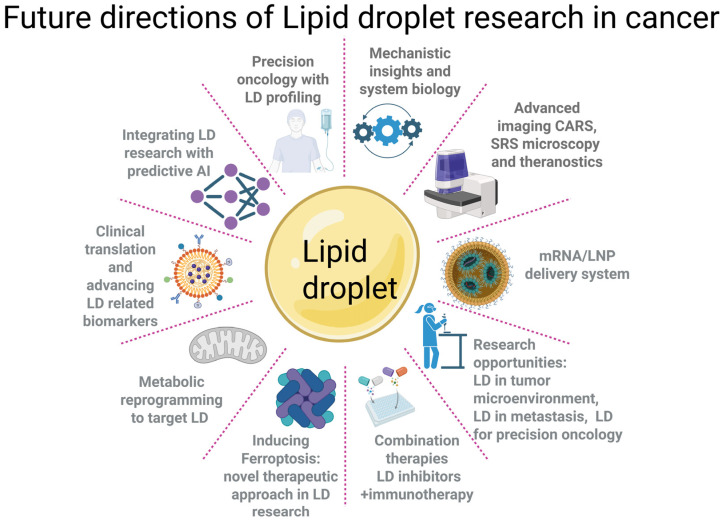
Schematic showing future directions of LD research in cancer.

Future research priorities include (Figure 1):

a. Selective Targeting: Identify cancer-specific LD regulators (e.g., PLIN2, SCD1, FABPs) and tumor-specific lipid compositions to design highly selective inhibitors.
b. Personalized Medicine: Incorporate LD profiling into clinical workflows to stratify patients based on metabolic phenotype. Tumors with high LD accumulation may respond better to FASN inhibitors, ferroptosis inducers, or LD-disrupting agents.
c. Advanced Technologies: Deploy single-cell lipidomics and spatial metabolomics to map LD heterogeneity and metabolic activity within the tumor microenvironment. These tools will reveal vulnerabilities and guide precision therapies.
d. Innovative Imaging and Delivery Platforms: Develop LD-targeted contrast agents for MRI/PET and engineer artificial LDs as drug delivery systems to selectively target lipid-rich tumors while minimizing systemic exposure.
e. Combination Strategies: Explore synergistic regimens combining LD-targeting drugs with immunotherapy or radiotherapy to overcome resistance and enhance efficacy.

In summary, advancing LD-targeted cancer therapy will require a multi-pronged approach: selective inhibitors, high-resolution lipidomics, innovative imaging and delivery platforms, and rational combination strategies. Coupled with robust biomarker development, these efforts will pave the way for precision oncology interventions that exploit LD biology to overcome drug resistance and improve patient outcomes (Figure 1).

## Figures and Tables

**Table 1:** Common methods for LD visualization.

Method	Principle	Examples	Technical Facilities	Pros	Cons
Colorimetric Staining	Lipophilic dyes bind to neutral lipids	Sudan III, Oil Red O	Light microscope; histology setup	Simple, economical, for fixed tissue sections only	Non-fluorescent; not appropriate for live-cell imaging
Fluorescence Microscopy	Fluorescent probes partition into LD core	Nile Red, BODIPY 493/503	Fluorescence microscope; filter sets	Enables live-cell imaging	Spectral crosstalk, poor photostability
Confocal Microscopy	Optical sectioning for 3D LD imaging	BODIPY variants	Confocal microscope, lasers, image analysis software	High-resolution 3D imaging, precise localization	Expensive, requires expertise, photobleaching risk
Live-cell Imaging	Real-time LD dynamics with fluorophores	BODIPY, chalcone probes	Live-cell, fluorescence/ confocal microscope	Tracks LD dynamics, physiological significance	Dye toxicity, photobleaching
Label-free Imaging	Vibrational or refractive index contrast	Raman microscopy, holotomography	Raman spectroscope; holotomography system	No dye artifacts; chemical specificity	High cost, complex instrumentation, lower throughput
Electron Microscopy (EM)	Ultrastructure visualization	No dyes (sample fixation)	TEM or SEM	Nanometer resolution, detailed morphology	Labor-intensive, fixed samples only
Mass Spectrometry Imaging	Direct chemical composition mapping	MALDI, SIMS	MS imaging systems	Molecular-level composition	Requires specialized equipment, low spatial resolution

## Data Availability

No new data were created or analyzed in this study.
